# Enhancing particle focusing: a comparative experimental study of modified square wave and square wave microchannels in lift and Dean vortex regimes

**DOI:** 10.1038/s41598-024-52839-1

**Published:** 2024-02-01

**Authors:** Ali Ashkani, Azadeh Jafari, Mehryar Jannesari Ghomsheh, Norbert Dumas, Denis Funfschilling

**Affiliations:** 1 School of Mechanical Engineering, Faculty of Engineering, University of Tehran, P.O. Box 11155-4563, Tehran, Iran; 2 ICube, UMR 7357–CNRS–Université de Strasbourg, 1, Cours des Cigarières, 67000 Strasbourg, France; 3 Present Address: Robert Frederick Smith School of Chemical and Biomolecular Engineering, Cornell University, Ithaca, NY 14853 USA; 4 ICube, UMR 7357–CNRS–Université de Strasbourg, 300 bd Sébastien Brant, 67412 Illkirch, France

**Keywords:** Fluid dynamics, Experimental particle physics

## Abstract

Serpentine microchannels are known for their effective particle focusing through Dean flow-induced rotational effects, which are used in compact designs for size-dependent focusing in medical diagnostics. This study explores square serpentine microchannels, a geometry that has recently gained prominence in inertial microfluidics, and presents a modification of square wave microchannels for improved particle separation and focusing. The proposed modification incorporates an additional U-shaped unit to convert the square wave microchannel into a non-axisymmetric structure, which enhances the Dean flow and consequently increases the Dean drag force. Extensive experiments were conducted covering a wide range of Reynolds numbers and particle sizes (2.45 µm to 12 µm). The particle concentration capability and streak position dynamics of the two structures were compared in detail. The results indicate that the modified square-wave microchannel exhibits efficient particle separation in the lower part of the Dean vortex-dominated regime. With increasing Reynolds number, the particles are successively focused into two streaks in the lift force-dominated regime and into a single streak in the Dean vortex-dominated regime, in this modified square wave geometry. These streaks have a low standard deviation around a mean value. In the Dean vortex-dominated regime, the location of the particle stream is highly dependent on the particle size, which allows good particle separation. Particle focusing occurs at lower Reynolds numbers in both the lift-dominated and lift/Dean drag-dominated regions than in the square wave microchannel. The innovative serpentine channel is particularly useful for the Dean drag-dominated regime and introduces a unique asymmetry that affects the particle focusing dynamics. The proposed device offers significant advantages in terms of efficiency, parallelization, footprint, and throughput over existing geometries.

## Introduction

The separation and concentration of particles and cells are key processes in the field of life sciences^1–7^. These procedures hold particular significance in various applications, with a primary focus on advancements in cell sorting, single-cell encapsulation for subsequent single-cell analysis^7^, and medical testing^6,8^. Such techniques are indispensable for isolating and identifying specific cell types, enabling meticulous examination at the single-cell level. In addition, these methodologies have vital medical diagnostic applications, where accurate and efficient particle separation is imperative, particularly in the isolation of circulating tumor cells (CTCs)^2,9,10^. The precise separation and concentration of particles and cells can benefit both fundamental life sciences research and diagnostic and therapeutic applications in the fields of personalized medicine and disease monitoring.

Particle separation in microfluidics is generally divided into active and passive groups based on the driving forces leading to separation. Active methods rely on an external force field^9^. This field can be an acoustic field^11^, a magnetic field^12,13^, an electric field such as in electrophoresis^14^, or dielectrophoresis^15,16^, an optical field or optical tweezers^17,18^, or a thermal field^19^. Passive techniques rely on hydrodynamic forces^20,21^. Some of the most prominent passive techniques include hydrodynamic filtration (HDF)^22^, pinched flow fractionation (PFF)^23^, deterministic lateral displacement (DLD)^24^, and inertial microfluidic^6,25,26^. Passive microfluidic devices are typically designed to take advantage of the inherent properties of fluids such as viscoelasticity^27^ or hydrodynamic forces generated by intrinsic channel geometries, such as inertial microfluidics. Active techniques provide excellent precision in cell manipulation by adjusting external forces, but typically operate at low throughput^6^. In contrast, inertial microfluidics is easy to use and is well suited for higher flow rates^28^.

Interest in inertial microfluidics has grown steadily since the pioneering work of Segré and Silberberg in 1961^25^. They reported the migration of small, neutrally buoyant spheres of different sizes in a circular tube to an equilibrium position on an annulus with a radius of 0.6 times the tube radius. The work of Di Carlo et al.^26^ in 2007 was another milestone in the development of inertial microfluidics for cell sorting and particle separation. By using symmetric and asymmetric serpentine channels, Di Carlo et al.^26^ were able to focus the particles into one or two streaks, depending on the flow rate. They were able to separate the particles because the location of the focus depends on the diameter of the particles. Their work has highlighted the potential of such techniques for medical applications and diagnostics, which currently require large volumes of blood, whereas a single drop of blood should be sufficient for such tests using microsystems^29,30^. Inertial microfluidics can concentrate the usually low concentration of pathogens in blood, which is an important point for early detection and prevention of diseases^31^. Macroscale cell separation techniques can lead to contamination and involve high accelerations, posing a potential threat to cell viability^32–34^. In contrast, microfluidics enable rapid and less aggressive separation of biological and synthetic particles and is immune to clogging, which is a common problem with physical filters^33,35^. Furthermore, their ease of fabrication and high portability enable the parallelization of microfluidic devices for low cost and efficient particle and cell separation^6,20^.

While inertial microfluidics is a well-established technique among passive particle separation methods, it only works at relatively high flow rates. Therefore, channel geometry is a critical design parameter that contributes to the device performance^6^. Channels with different cross-sections have been studied: (i) straight microchannels with square cross-sections^36–38^, (ii) rectangular cross-sections^39^, (iii) triangular cross-sections^40^ (iv) curved cross-sections^41^, (v), and trapezoidal cross-sections^35^.The microchannels used for inertial microfluidics also have different geometries: (i) straight microchannels^42,43^, (ii) spiral microchannels^44,45^, (iii) curved microchannels^26,46^, (iv) expansion–contraction microchannels^2,47^, (v) serpentine microchannels^5,6,48^, (vi) asymmetrically curved microchannels^4,34^, (vii) zigzag microchannels^48^, (viii) square wave microchannels^32,48^, (ix) trapezoidal straight microchannels^35^, and (x) wavy microchannel structures^31^. For a more comprehensive review of inertial microfluidics, see^49^ or^10^. Focusing depends on the balance between the different forces acting on the particles. In the case of a straight microchannel, these forces are: (1) Saffman force: The presence of a wall creates a velocity gradient in the fluid and leads to shear-induced particle rotation. The additional drag created by the walls causes the particle to lag behind the fluid. This slip-shear motion generates a lateral force on the particles called the Saffman force^49,50^, (2) the Magnus force, a rotation-induced lift force^49,51^, (3) the wall-induced lift force^48,52^, and (4) the shear gradient lift force resulting from the curvature of the velocity profile^6,48,49,52,53^. The Saffman and Magnus forces are often very small and negligible for particle motion in a channel flow^49,52^. The dominant forces for the lateral migration of the particles are the wall-induced lift force, which pushes the particles away from the wall, and the shear gradient lift force, which pushes the particles away from the channel centerline in a Poiseuille flow^48,49,52^. The equilibrium between the wall lift force and the shear gradient lift force creates multiple equilibrium positions between the channel walls and the centerline.

In the case of curved channels, i.e., serpentine, spiral, triangular, curved, or contraction–expansion microchannels, the curvature of the geometry leads to a secondary rotational flow, called Dean flow, caused by the pressure gradient in the radial direction^54^. Di Carlo^1^ demonstrated the role of inertial lift and Dean drag forces in the final equilibrium position of particles in microchannels. Particles flowing in a channel of a given geometry can be focused depending on the relative importance of the lift and Dean drag forces exerted on the particles^55^. The main variables that control particle focusing are the flow rate, particle size^6^, and channel dimensions^56^. Dean flow induces a stirring effect that accelerates the lateral migration of particles to their equilibrium positions, thereby reducing the required channel length^10^. Inertial lift and Dean drag forces scale differently with particle size, resulting in size-dependent focusing positions that allow the separation of particles of different sizes^6,10^.

In recent years, serpentine microchannels have become increasingly popular in inertial microfluidics to generate Dean flow^5,57^ because they improve particle focusing^58^ while reducing the microchannel footprint compared to spiral microchannels for the same throughput^59^. More specifically, a chalenge in inertial microfluidics is achieving the maximum throughput at a given footprint. This can be achieved by parallelizing the microchannels^60^. One limitation of spiral microchannels is the complexity involved in arranging several of these systems on a single substrate^1^. However, the linear nature of serpentine microchannels allows for massive parallelization to increase the throughput per footprint^5^. Furthermore, the channel radius must increase with each turn in spiral channels, whereas serpentine channels consist of a series of alternating right-angle turns. The constant radius of serpentine channels allows for a smaller footprint than that of spiral channels^61^. In addition, in spiral channels, the Dean flow is generated in a single direction, whereas in serpentine channels, the direction of the Dean flow changes with each turn. The changing direction of the Dean flow in serpentine channels enhances the focusing of particles and cells^44^. Zhang et al.^32^ studied the focusing pattern of particles of different sizes in a serpentine microchannel for channel Reynolds numbers up to 200. Tang et al.^52^ presented innovative geometries for inertial focusing in microchannels.

Geometry optimization has recently become an active field of research^6,48^. Cha et al.^6^ investigated the effect of concave and convex obstacles in a sinusoidal channel on particle separation. Other studies have focused on the geometry, comparing serpentine, zigzag, and square microchannels^48^, or on the aspect ratio of the channel cross-section^46^ for the best separation of particles of different sizes and the shortest focusing distance. A shorter focusing distance means lower flow resistance and thus lower pressure and power required to drive the flow^55^. The design of the channel, i.e., the geometry of the cross-section of the channel and the geometry of the channel itself (triangular, serpentine, asymmetric curves, etc.), are also parameters that need to be optimized to limit pressure losses^48^, since pressure loss is the limiting factor for increasing throughput, as high pressure leads to leakage at the fluid inlets^55^.

In this study, the efficiency of two geometries is investigated and compared: a square wave geometry and a newly proposed modified square wave microchannel specifically designed for particle sorting. The manuscript is organized as follows. In the next section, a summary of the theory of inertial focusing is presented, followed by experimental details of the experimental setup and conditions. In the following section, experiments are performed over a large range of Reynolds numbers and particle sizes. The critical Reynolds number is then determined. An analysis of the focusing quality leads to a comparison of the efficiency of the two geometries. The conclusion is given in the last section.

As the Reynolds number increased, we observed distinct particle focusing patterns: two streaks in the lift-dominated regime, merging into a single streak in the Dean vortex-dominated regime. In particular, the square wave microchannel exhibited a specific transition behavior. Conversely, the modified square wave microchannel, with its inherently asymmetric geometry, exhibited asymmetric particle focusing, resulting in the gradual replacement of one flow by another during the lift/Dean vortex transition. To quantitatively compare the two geometries, we constructed histograms showing the particle distribution across the channel at different Reynolds numbers and particle sizes. The modified square-wave microchannel outperformed the Dean drag-dominated regime, exhibiting a size-dependent sweet spot for particle separation with a reduced standard deviation around the mean. Our results highlight the unique performance characteristics of the proposed geometry and provide insight into the optimization of particle separation, particularly in the Dean drag force-dominated regime.

## Theory of inertial focusing

Inertial microfluidics operates at an intermediate Reynolds number, i.e. 1 < Re < 100^6,52^, where both fluid inertia and viscosity are finite. Finite fluid inertia leads to several intriguing inertial effects that form the basis of inertial microfluidics. These include: (i) inertial migration and (ii) secondary flow.

Inertial migration is a phenomenon in which randomly dispersed particles at the entrance of a channel migrate laterally to multiple equilibrium positions after a sufficient distance. For a neutrally buoyant rigid sphere flowing in a straight wall-bounded Poiseuille flow, four lateral forces act on the sphere in addition to a viscous drag force: (1) Magnus force due to slip rotation, (2) Saffman force due to slip shear, (3) wall lift force due to the disturbance of the flow field around particles by the wall, and (4) shear gradient lift force due to curvature of the undisturbed fluid velocity profile. Among them, The Magnus and saffman forces are often very small and negligible. Shear gradient lift force, which directs particles toward the channel walls, and wall lift force, which repels particles toward the channel centerline, are generally recognized as the dominant effects on the lateral migration of particles. The balance between the shear gradient lift force and the wall lift force creates multiple equilibrium positions midway between the channel walls and the centerline.

**Figure 1:**
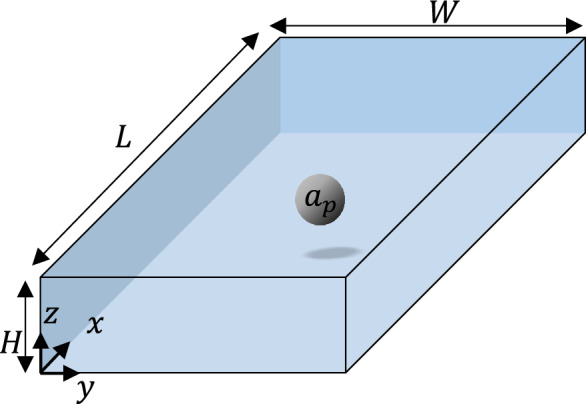
Schematic of a straight microchannel with a single particle.

The total inertial lift force in a straight channel is a function of the microchannel geometry, particle diameter, and velocity profile shape profile. It can be expressed as:

1 $$F_{L} = F\left( {a_{p} ,y,z,H,W,V_{c} ,\mu ,\rho_{f} } \right)$$

where ${a}_{p}$ is the particle diameter, $y$ and $z$ are the positions of the center of the particle in the microchannel cross-section (see Fig. 1), ${V}_{c}$ is the characteristic velocity of the fluid, ${\rho }_{f}$ is the fluid density, and $\mu$ the fluid viscosity. It is common to consider the maximum velocity as the characteristic velocity. Using the Buckingham-Pi theorem, the dimensionless form of the inertial lift force is:

2 $${F}_{L}^{*}=F (\kappa ,{y}^{*},{z}^{*},AR,{Re}_{c})$$

where $\kappa =\frac{{a}_{p}}{H}$ is the blockage ratio, ${y}^{*}=\frac{y}{W}$ and ${z}^{*}=\frac{z}{H}$ are the non-dimensional position of the particle, $AR$ is the aspect ratio of the microchannel, and $R{e}_{c}$ is the channel Reynolds number of the flow, generally defined as $R{e}_{c}=\frac{{\rho }_{f}{V}_{c}{L}_{c}}{\mu }$, where ${L}_{c}$ is the characteristic length. In a rectangular microfluidic channel, the hydraulic diameter ${D}_{h}=\frac{2WH}{\left(W+H\right)}$ is usually considered to be the characteristic length ${L}_{c}$ and the characteristic velocity of the fluid is considered to be the mean flow velocity, ${U}_{m}$. In addition to the channel Reynolds number, the particle Reynolds number characterizing the particle flow, ${Re}_{p}={Re}_{c}\times {\left(\frac{{a}_{p}}{{D}_{h}}\right)}^{2}$ is an important non-dimensional number in microfluidic channels^1,26^.

Two conditions are required for particle focusing: (i) the ratio (particle size) to (hydraulic diameter)—often referred to as the particle-blockage ratio—must exceed a critical ratio to achieve particle focusing, i.e. $\lambda ={a}_{p}/{D}_{h}>0.07$^5,26,28,48^ and (ii) the particle Reynolds number must also exceed one, i.e. ${Re}_{p}={Re}_{c}\times {\left({a}_{p}/{D}_{h}\right)}^{2}>1$^26,48^. Some authors, such as^62^, were more detailed about the critical value of the particle blockage ratio and observed different focusing modes: (1) focusing mode for $\lambda >0.07$, (2) non-focusing mode for $\lambda <\sim 0.01,$ in this mode the particle dynamics are dominated by Brownian motion, and (3) ‘rough’ focusing mode in between, where particles can migrate to form a relatively large particle band. Miller et al.^63^ gave a different empirical relationship for the critical aspect ratio $\lambda >0.021356\times {H}^{1.33623}$ where $H$ is the channel height in µm.

### Lift forces

The main forces acting on the particles flowing in a curved channel and inducing focusing are the shear gradient lift force ${F}_{LS}$, the wall-induced lift force ${F}_{LW}$ and Dean drag forces^48,52^. As is usual in microfluidics, the gravitational force on the particles is neglected. The wall-induced lift force ${F}_{LW}$ directs the particles away from the wall due to pressure imbalance caused by the asymmetric wake behind the particle, whereas the shear gradient lift force, ${F}_{LS}$, directs the particles away from the channel center in Poiseuille flow due to the profile of the velocity field, leading to a pressure difference around the particle^48,52^. These forces can be expressed as follows:

3 $${F}_{LS}=\frac{{f}_{LS}{\rho }_{f}{U}_{m}^{2}{{a}_{p}}^{3}}{{D}_{h}}$$

4 $${F}_{LW}=\frac{{f}_{LW}{\rho }_{f}{U}_{m}^{2}{{a}_{p}}^{6}}{{D}_{h}^{4}}$$

where ${U}_{m}$ is the mean flow velocity and ${f}_{LS}$ and ${f}_{LW}$ are the shear gradient and wall-induced lift force coefficients, respectively. A unique expression of inertial lift forces that combines both wall-induced and shear gradient lift forces is often used^1,6,48,52,55^:

5 $${F}_{L}=\frac{{f}_{L}{\rho }_{f}{U}_{m}^{2}{{a}_{p}}^{4}}{{D}_{h}^{2}}$$

where ${f}_{L}$ is a dimensionless lift coefficient that is a function of the particle position across the channel cross-section. In a straight channel, by balancing the inertial lift with the Stokes drag $\left({F}_{St}=3\pi \mu {a}_{p}{U}_{L}\right)$, where ${U}_{L}$ is the lateral migration velocity of the particles, this migration velocity can be expressed as^26,28^:

6 $${U}_{L}=\frac{{f}_{L}{\rho }_{f}{U}_{m}^{2}{{a}_{p}}^{3}}{3\pi \mu {{D}_{h}}^{2}}$$

Zhou and Papautsky^28^ experimentally measured the value of these inertial forces by measuring the particle migration distance ${L}_{m}$ in a channel of length $L,$ $L={U}_{m}{L}_{m}/{U}_{L}$. From these measurements, they derived an expression for the lift force coefficient that has been widely used^6^:

7 $${f}_{L}\sim \frac{1}{\sqrt{{Re}_{c}}}{\left(\frac{{D}_{h}}{{a}_{p}}\right)}^{2}$$

where $${Re}_{c}=\frac{{\rho }_{f}{U}_{m}{D}_{h}}{\mu }$$.

In a straight channel, Zhou and Papautsky^28^ described the focusing of the particles in two steps. In the first step, the shear-induced lift is the leading force that drives the particles toward the channel wall. In a much slower second step, as the particle approaches the wall, the wall-induced lift force grows, balances the shear-induced lift force, and becomes dominant, resulting in a net force along the wall toward its center.

### Dean drag force

Secondary flow usually occurs in a curved channel or a straight channel with obstructions. In a curved channel, secondary flow is induced by a pressure gradient in the radial direction due to a fluid momentum mismatch between the center and near-wall regions within the curvature. Thus, fluid elements near the channel centerline flow outward and push the relatively stagnant fluid elements near the channel wall inward along the circumference, forming two counter-rotating flows called Dean vortices. Introducing this secondary flow into inertial focusing has several advantages. For example, the Dean vortex can modify the inertial equilibrium positions by imposing an additional viscous drag force on particles perpendicular to the main flow. Size-dependent differential focusing of particles according to the ratio of inertial lift to secondary flow drag (F_L_/F_D_) promises complete particle separation. In addition, the Dean vortex could reduce the channel length/footprint due to the mixing effects of the secondary flow, helping the particles to reach the equilibrium position more quickly.

In a curved channel, Dean vortices are present because the fluid velocity in the center is faster than that near the wall, which induces a pressure gradient along the radial direction and results in two counter-rotating vortices named Dean vortices. The Dean number is expressed as^55^:

8 $$De=\sqrt{\frac{{D}_{h}}{2R}}{Re}_{c}$$

where R is the radius of curvature of the channel. The velocity magnitude of the secondary flow induced by the Dean vortex scales is^1,6,26,55^:

9 $${U}_{D}\sim \frac{{\mu De}^{2}}{{{\rho }_{f} D}_{h}}$$

Other estimates exist, such as the one of Özbey et al.^4^ who estimated the average Dean velocity to be ${U}_{D}=1.8\times {10}^{-4}{De}^{1.63}$. Based on the Stokes’ law, the magnitude of the Dean drag force, ${F}_{D}$ scales as^6,26^:

10 $${F}_{D}\sim \frac{{\mu }^{2}{a}_{p}{De}^{2}}{{\rho }_{f}{D}_{h}}$$

### Inertial lift vs. Dean drag

The balance between the inertial lift (${F}_{L}$) and Dean drag forces (${F}_{D}$) determines the preferred location of the focusing positions. Dean drag forces do not cause particle focusing but act in superposition with the inertial lift forces to reduce the number of equilibrium positions created by the inertial lift forces.

In this particular study, we focused on the serpentine channel. Unlike in a spiral channel, In a serpentine channel, curvature alternates periodically, and thus the secondary flow may not approach a steady state after each turn. Similarly, the movement of particles may also not reach steady state, and the accumulation of this unsteadiness may cause unpredictable and non-intuitive behavior of particles.

The inertial focusing and equilibrium positions in a curved channel are the result of the competition between the inertial lift and Dean drag forces^6,55^. Therefore, the relative ratio between these forces ${F}_{L}/{F}_{D}$ has often been estimated as:

11 $$\frac{{F}_{L}}{{F}_{D}}\sim \frac{{R{ a}_{p}}^{3}}{{{D}_{h}}^{4}}{f}_{L}$$

which, using the lift force coefficient measured experimentally by^28^ in a straight channel, becomes

12 $$\frac{{F}_{L}}{{F}_{D}}\sim \frac{R {a}_{p} }{{{D}_{h}}^{2.5}{U}_{m}^{0.5}}$$

In the lift force dominated-regime, ${F}_{L}\gg {F}_{D}$, i.e., inertial forces dominate and particles migrate to the equilibrium position of the inertial lift forces. In the Dean-vortex dominated regime, ${F}_{L}\ll {F}_{D}$, i.e. Dean drag force dominates and particles follow the line of the center or near the center of the Dean vortices (see Jiang et al.^64^). In between, the two forces are of the same order of magnitude, and the inertial focusing positions can be modified by the Dean flow. Secondary flow in curved channels is the dominant factor contributing to the modified equilibrium positions for particles flowing at finite Reynolds numbers^55^. Di Carlo et al.^26^ gave a slightly different expression for this relationship

13 $$\frac{{F}_{L}}{{F}_{D}}\sim \frac{1}{\delta }{\left(\frac{{a}_{p}}{{D}_{h}}\right)}^{3}{{Re}_{c}}^{n} \left(n<0\right)$$

where $\delta ={D}_{h}/2R$, R is the radius of curvature. $\frac{{F}_{L}}{{F}_{D}}=\frac{{{a}_{p}}^{2}R}{{H}^{3}}$ is another expression given by^1^ with a threshold value of 0.04 ($H$ is the smallest channel dimension). Using particles of different densities, Di Carlo et al.^26^ observed that inertia had no effect on the focusing of particles in a single line and concluded that the focusing on a single line was due to the Dean vortex and not to inertial shear lift or wall lift forces. The idea was not completely clear in the community; some authors believed that for high Reynolds numbers, the inertial forces become dominant over the secondary flow effect (i.e. over the Dean drag forces)^2^, while a large majority agreed with the Di Carlo statement.

When the straight channel has a square cross-section, the inertial migration of cells or particles results in focusing at four equilibrium positions centered on the faces of the particles^28^. In the case of a channel with a rectangular cross-section, the number of equilibrium positions is reduced to two^33^. The aspect ratio of the channel cross-section (height over width) has a large influence on focusing^5,44,65^. In most experimental studies, the aspect ratio of the microchannel is often 2 or more.

Blahout et al.^21^ measured the three-dimensional particle distribution in a serpentine channel with sharp corners using particle tracking velocimetry. The equilibrium trajectories of particles develop not only over the channel width, but also over the channel height. In their Reynolds numbers range ($100<Re<150$), 9.87 µm particles were focused in two equilibrium positions on top of each other, close to the channel bisector, while 3.55 µm particles were focused in four equilibrium positions, forming two pairs of particles on top of each other, located approximately halfway between the channel center and the wall. These 4 equilibrium positions exist up to a critical Reynolds number^21^. Above this critical Reynolds number, the particles were concentrated in two equilibrium positions. Looking at the microchannel from above, as in most studies, the four and two focusing positions vertically overlap in pairs and thus appear as two and one lines, respectively. Thus, the observations of Di Carlo^1^ and Blahout et al.^21^ are in full agreement with those of Di Carlo et al.^26^, Zhang et al.^32^, and Ying and Lin^48^.

The numerical simulations of Jiang et al.^64^, also performed in a serpentine microchannel, confirmed the four and two equilibrium positions of Blahout et al.^21^. For them, at lower Re, lift forces were also dominant and particles focus on two lines. Small particles are found to focus closer to the sidewalls than large particles. The migration of particles toward the inertial equilibrium positions is facilitated by secondary flow, which sweeps particles toward the sidewalls^54^. At reasonably high Re, the Dean drag force is enhanced, and the Dean flow swings the particles out of the trap of the lift forces and concentrates the particles in the horizontal center of the channel near the Dean flow vortex center^64^. In addition, large particles tend to rotate with the Dean flow and flow to the channel center ahead of the small particles, allowing the separation of particles of different sizes. Pedrol et al.^65^ confirmed that inertially focused particles are confined within the Dean vortex centerlines.

## Experimental framework for microsystem design and particle analysis

### Microsystem fabrication

The main part of the setup consists of microfluidic microsystems with specific geometries. The microsystem was produced with the so-called soft lithography technique using polydimethylsiloxane (PDMS). In brief, a mask with specific geometries was printed on a slide with a high-resolution inkjet printer (SELBA, Versoix, Switzerland). A uniform layer of SU8 resin (a permanent negative epoxy photoresist resin, MicroChem, Newton MA, USA) of approximately 50 µm was applied on a 3-in. silicon wafer by first pouring the resin on the wafer and then using a spinner to obtain a thin uniform layer.

After the baking step, the SU8 resin is polymerized locally by applying parallel UV light through the mask. After development, the wafer is covered with a uniform layer of PDMS (Sylgard™ 184 Dow Chemical). PDMS is prepared by mixing PDMS oil with a curing agent in a ratio of 1 to 10. The mixture is placed under vacuum for 3 h to extract the inserted bubbles first in a beaker and then on the SU8 Master. The final step is to cure the PDMS by heating it in an oven at 65 °C for 1 h. Later, the now elastomeric PDMS is peeled off the wafer and bonded to a microscope glass plate by the oxygen plasma bonding technique.

### Microsystem geometries

**Figure 2:**
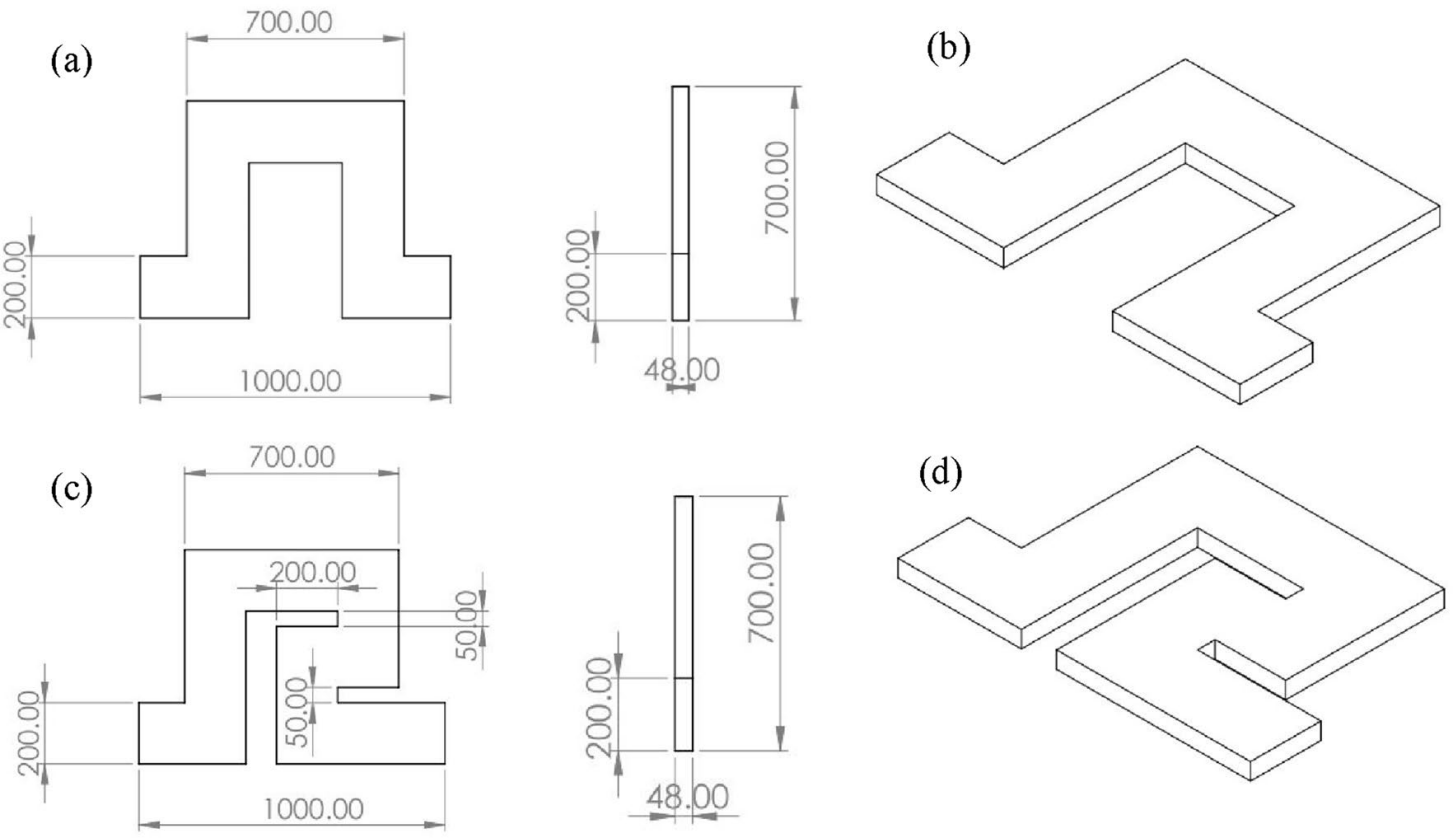
Geometries of the two microchannels used in this study. (**a**) Top and side views and (**b**) 3D sketch of a unit of the “square wave” microchannel, (**c**) Top and side views and (**d**) 3D sketch of a unit of the “modified square wave” microchannel.

**Figure 3:**
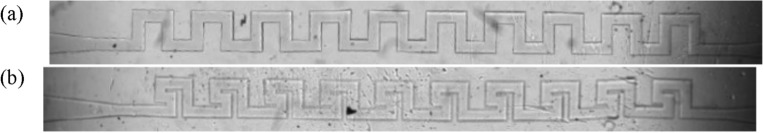
Images of the (**a**) square wave microchannel and (**b**) modified square wave microchannel.

In this study, we used two different microsystem geometries, the first being a “square wave” microchannel already used by Zhang et al.^32^ and the second being a “modified square wave” microchannel proposed by us to improve footprint and throughput. The channel depth is 48 µm, and the width is 200 µm. Both geometries are shown in Figs. 2 and 3. In inertial microfluidics, microchannels often have a rectangular cross-section with a low aspect ratio, i.e., a width-to-height ratio of 2 or less, because it promotes focusing^46,55^.

### Particle preparation

Particles with diameters between 2.45 and 12 µm were used in the experiments (see Table 1). These particles are calibrated polystyrene particles (sometimes called Latex particles), most of the time monodispersed in water, typically at a concentration of 5 wt% in water.

**Table 1:** Particles used for Dean and Lift force focusing.

Particle size [µm]	$\lambda ={a}_{P}/{D}_{H}$	Name	Manufacturer	Characteristics
1	0.013	K1-100	Estapor^®^	Microsphere latex calibrated particles, diameter 1.009 µm
2.45	0.032	L1-200	Estapor^®^	Microsphere latex calibrated particles, diameter 2.45 µm
4.16	0.054	R09-11	Estapor^®^	Microsphere latex calibrated particles, diameter 4.16 µm
5.9	0.076	CML Latex, 4% w/v 6 µm	Invitrogen by Thermo Fisher Scientific	Actual size 5.9 µm, concentration 4.0 g/100 ml
8	0.103	84,192-5 ml-F	Sigma-Aldrich	Microparticles size standard based on polystyrene monodispersed, 8 µm
10	0.129	61,946-5 ml-F	Sigma-Aldrich	Microparticles based on polystyrene, dark red, 10 µm
12	0.155	88,511-5 ml-F	Sigma-Aldrich	Microparticles size standard based on polystyrene monodispersed, 12 µm

These particles were diluted in distilled water to reach a concentration of 0.05% weight ratio (similar to the concentration used by Zhang et al.^32^).

### Experimental setup and image analysis

A high-precision syringe pump system, NEMESYS (Cetoni GmbH, Korbussen Germany), was used, and the particle flow was imaged on a Nikon Eclipse TS100 inverted microscope with a Nikon LWD 20×/0.40 objective and a Miro 110 Lab high-speed camera (Phantom Vision Research Inc., USA). Particles are imaged using backlight illumination, where a strong LED light (Schott KL 2500 LED, Mainz, Germany) illuminates the microsystem on the opposite side of the microscope objective, so that the particles appear black on a white background.

The channel flow rate was varied between 50 µL/min and 700 µL/min by 50 µL/min steps with a step refinement of 25 µL/min in the transition region between the lift force-dominated regime and the Dean-vortex dominated regime. By increasing the flow rate, we first meet the lift force-dominated regime and then the Dean vortex dominated-regime. The channel Reynolds number,${Re}_{c}=\frac{{\rho }_{f}{U}_{m}{D}_{h}}{\mu }$ , i.e., the dimensionless number built on the ratio of inertial and viscous forces, was used as the parameter characterizing the flow regime.

$\rho = 1000 \;\; \mathrm{kg}/{{\text{m}}}^{3}$ is the fluid density (water density), Um is the fluid velocity [m/s], ${D}_{h}= 77.4$ µm is the hydraulic diameter, $\mu =0.001$ Pa s is the viscosity of the fluid considered as the viscosity of water. The channel Reynolds number, particle Reynolds number, and Dean number, of the different experiments are given in Table 2.

**Table 2:** Experimental conditions for the study of different particle flows.

Flow rate $\left[\frac{\upmu\text{L}}{\text{min}}\right]$	Mean velocity $\left[\frac{\text{m}}{\textrm{s}}\right]$	Channel’s Re number $\left(R{e}_{c}\right)$	Dean number $\left(De\right)$	Particle’s Re number $(R{e}_{p})$					
				${a}_{p}=1$ μm	${a}_{p}=2.45$ μm	${a}_{p}=4.16$ μm	${a}_{p}=5.9$ μm	${a}_{p}=8$ μm	${a}_{p}=12$ μm
$50$	$0.0868$	$6.7188$	$4.2118$	$0.0011$	$0.0067$	$0.0194$	$0.0390$	$0.0718$	$0.1615$
$100$	$0.1736$	$13.4375$	$8.4235$	$0.0022$	$0.0135$	$0.0388$	$0.0781$	$0.1436$	$0.3230$
$150$	$0.2604$	$20.1563$	$12.6353$	$0.0034$	$0.0202$	$0.0582$	$0.1171$	$0.2153$	$0.4845$
$200$	$0.3472$	$26.8750$	$16.8470$	$0.0045$	$0.0269$	$0.0776$	$0.1562$	$0.2871$	$0.6460$
$250$	$0.4340$	$33.5938$	$21.0588$	$0.0056$	$0.0337$	$0.0970$	$0.1952$	$0.3589$	$0.8075$
$300$	$0.5208$	$40.3125$	$25.2705$	$0.0067$	$0.0404$	$0.1165$	$0.2342$	$0.4307$	$0.9690$
$350$	$0.6076$	$47.0313$	$29.4823$	$0.0079$	$0.0471$	$0.1359$	$0.2733$	$0.5024$	$1.1305$
$400$	$0.6944$	$53.7500$	$33.6940$	$0.0090$	$0.0539$	$0.1553$	$0.3123$	$0.5742$	$1.2920$
$450$	$0.7813$	$60.4688$	$37.9058$	$0.0101$	$0.0606$	$0.1747$	$0.3514$	$0.6460$	$1.4535$
$500$	$0.8681$	$67.1875$	$42.1175$	$0.0112$	$0.0673$	$0.1941$	$0.3904$	$0.7178$	$1.6150$
$550$	$0.9549$	$73.9063$	$46.3293$	$0.0123$	$0.0741$	$0.2135$	$0.4294$	$0.7895$	$1.7765$
$600$	$1.0417$	$80.6250$	$50.5410$	$0.0135$	$0.0808$	$0.2329$	$0.4685$	$0.8613$	$1.9380$
$650$	$1.1285$	$87.3438$	$54.7528$	$0.0146$	$0.0875$	$0.2523$	$0.5075$	$0.9331$	$2.0995$
$700$	$1.2153$	$94.0625$	$58.9646$	$0.0157$	$0.0942$	$0.2717$	$0.5466$	$1.0049$	$2.2610$
$750$	$1.3021$	$100.7813$	$63.1763$	$0.0168$	$0.1010$	$0.2911$	$0.5856$	$1.0767$	$2.4225$
$800$	$1.3889$	$107.5000$	$67.3881$	$0.0179$	$0.1077$	$0.3105$	$0.6246$	$1.1484$	$2.5840$

Between 2000 and 6000 images were taken at a rate of 100 frames per second for each flow rate and particle size. The images were analyzed using programs developed with the software MATLAB (MathWorks, Natick Massachusetts USA).

**Figure 4:**
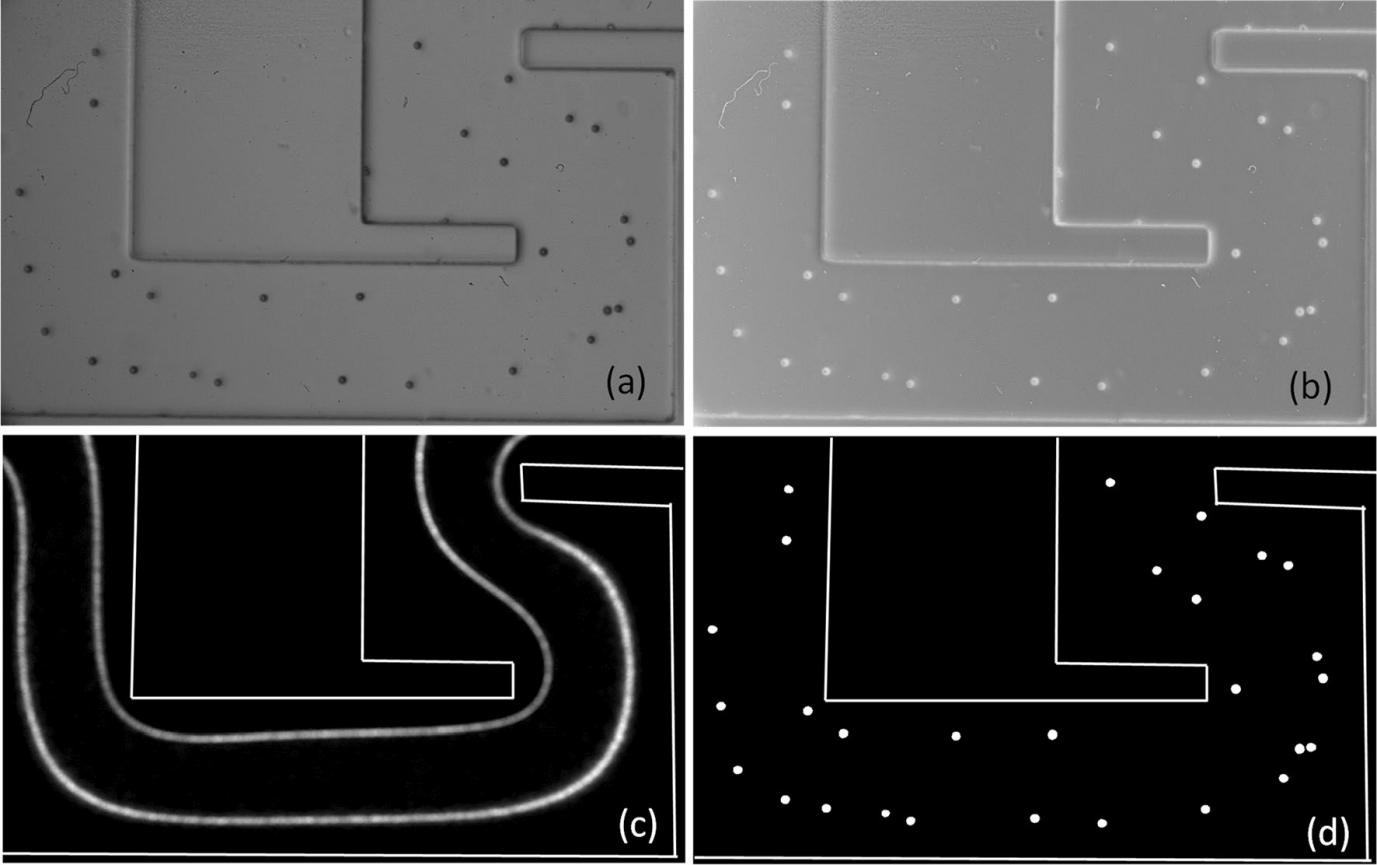
(**a**) ordinal image, (**b**) inverted image, (**c**) mean stream of the particle, and (**d**) black and white image of the particles.

The goal of the first program was to obtain a qualitative picture of the effectiveness of different geometries on focusing particles and to determine the effect of particle size. In the ordinal image, particles appear black on a white background because of to backlight illumination (see Fig. 4a). These tiff8 images were inverted (see Fig. 4b), so that the particles now appear white on a black background. By comparing these inverted images (Fig. 4b) with the inverted mean image, and by applying a threshold of 20 gray levels to the 256 (i.e. 28) byte images for particle detection, a cumulative image showing the particle stream was obtained (see Fig. 4c). For better visualization, Fig. 4c was rescaled for each series to avoid saturation, which would result in loss of information.

A second program detects and locates the particles individually, so that statistics on the position of the particle on a cross-section can be obtained from the 2000 or 6000 images. A threshold is applied to the inverted image (Fig. 4b) to isolate the particles from the background, resulting in binary black and white images (see Fig. 4d), which are easier to use for image processing.

## Results and discussion

In this section, the experimental results for both the square wave and the modified square wave microchannels are obtained over a large range of Reynolds numbers [6.7 ≤ Re ≤ 107.5] and particle sizes ranging from 2.45 to 12 μm. A comprehensive study of the focusing quality at different Reynolds numbers and particle sizes is performed to determine the critical Reynolds number for both the lift and Dean dominated regimes.

### Flow field at low Reynolds number

Particles of 1.0 µm were used for particle image velocimetry (PIV) flow field measurements. These particles are small enough to follow the flow, but large enough to neglect Brownian motion, which could interfere with flow field measurements. In fact, the diffusion coefficient is $D=\frac{RT}{3\pi \mu {a}_{p}{N}_{A}}=2.14\times {10}^{-13} \; {\text{m}}^{2}/{\text{s}}$, where *R* is the universal gas constant , $T$ the temperature, $\mu$ the viscosity, ${a}_{p}$ the particle diameter, and *N*_A_ is the Avogadro constant. The diffusion time is ${\tau }_{diffusion}={a}_{p}^{2}/(4D)= 4.66\; \text{s}$, and considering the slowest flow velocity in our experiment (i.e., 0.09 m/s), the convection time is ${\tau }_{convection}={a}_{p}/(2U)=1.1\times {10}^{-5} \; \text{s}$ therefore, ${\tau }_{diffusion }\gg {\tau }_{convection}$ and Brownian motion is negligible.

The images were taken at the highest frequency allowed by our high-speed camera (i.e., 2000 fps). After dividing of the images by a mean image to minimize the effect of illumination defects and the not so uniform illumination of the raw image, the flow field was calculated by cross-correlation between successive images using the particle image velocimetry software PIVlab (open source software on MATLAB written by William Thielicke, https://pivlab.blogspot.com/).

**Figure 5:**
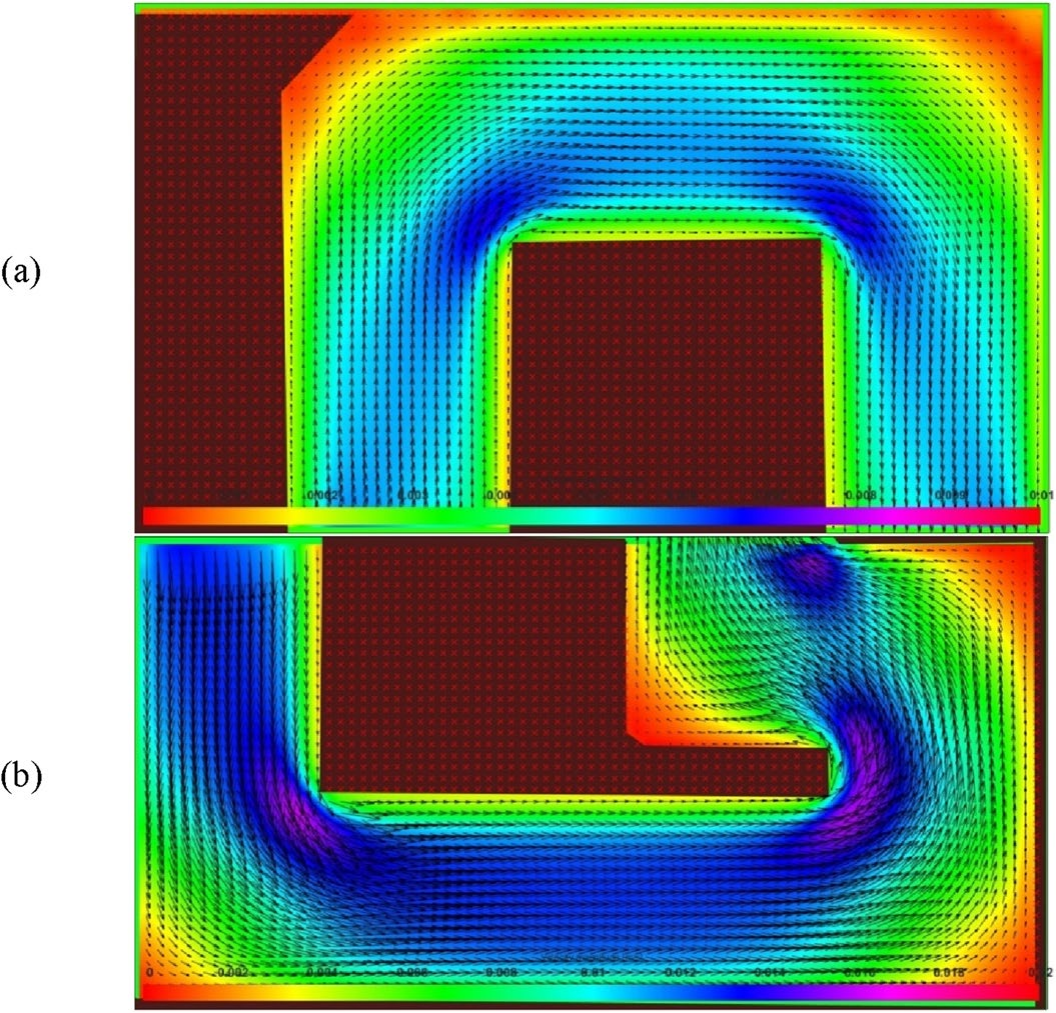
Flow field on the horizontal plane of symmetry of (**a**) the square wave and (**b**) the modified square wave microchannels. The flow field is measured for Reynolds numbers of 0.3 and 0.67. The velocity scale bar is in m/s.

The flow field (Fig. 5) was measured in the horizontal plane of symmetry of the channels for Reynolds numbers smaller than those where the effects of the lift forces and Dean drag force are observed. However, because the Reynolds numbers remain laminar in this study (see Table 2, the highest Reynolds number is 110), and because the Dean vortex appears only as a secondary recirculation and is located on a cross-section of the channel, the main flow field is qualitatively similar for all the Reynolds numbers.

### Effect of Reynolds number and particle size

**Figure 6:**
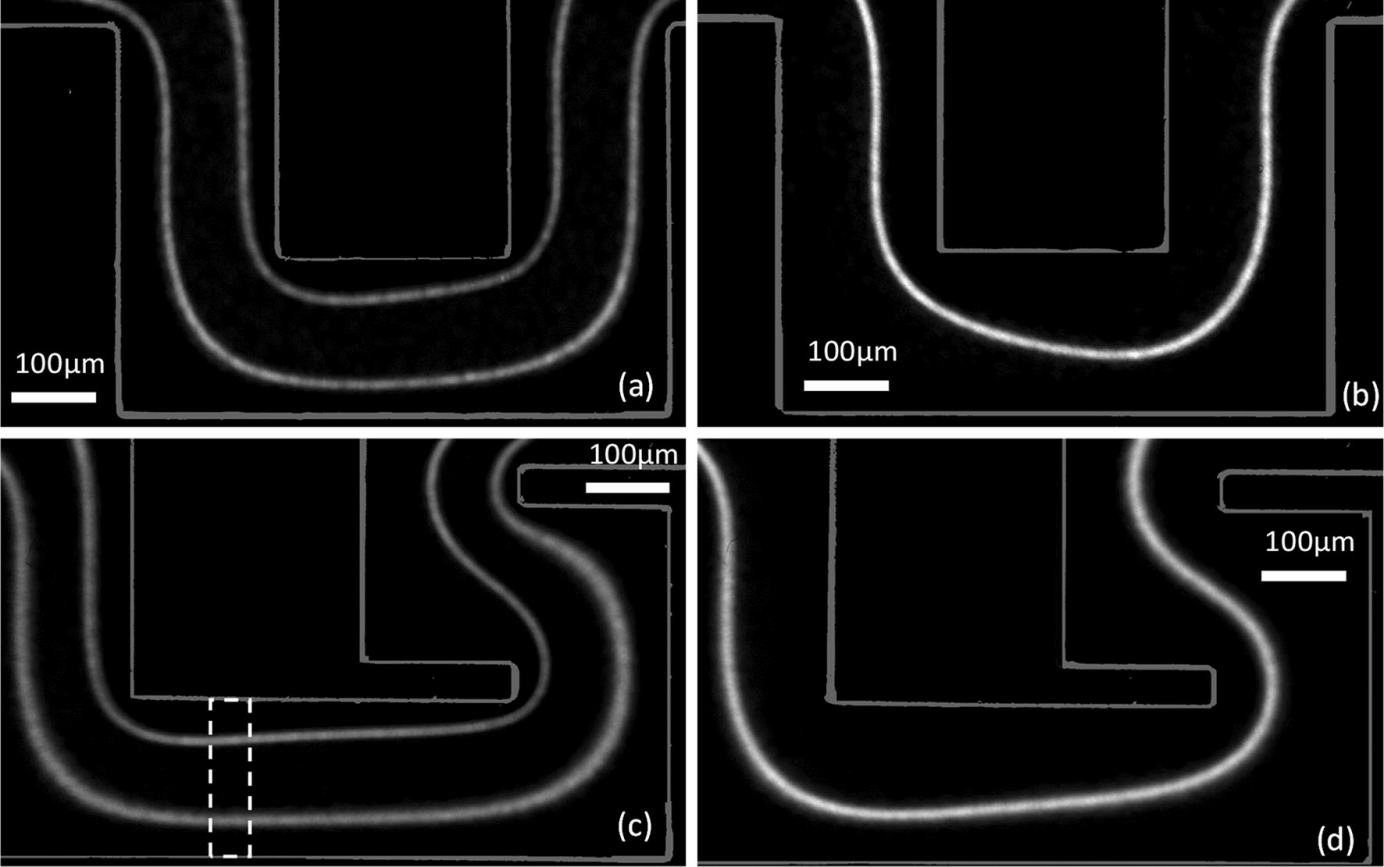
Mean trajectory of 8 µm particles for (**a**) the square wave microchannel, ${{\text{Re}}}_{{\text{c}}}=40.2$, (**b**) the square wave microchannel, ${{\text{Re}}}_{{\text{c}}}=77.3$, (**c**) the modified square wave microchannel , ${{\text{Re}}}_{{\text{c}}}=33.6$, (**b**) the modified square wave microchannel, ${{\text{Re}}}_{{\text{c}}}=$ 67.2.

Four steps can be distinguished in the evolution of particle focusing with increasing Reynolds number: (i) in the lift forces-dominated regime (relatively low Reynolds numbers), particles are aligned on two streams (see Fig. 6a and c), located symmetrically around the center of the channel as in Zhang et al.^32^, (ii) with increasing Reynolds number, the Dean drag force becomes strong enough to compete with the inertial lift forces and modify the lift force focusing (7, 12). We enter the transition zone. The length of this transition zone depends on the particle sizes and microchannel structure, (iii) above a critical Reynolds number, we enter the Dean drag force-dominated regime where the particles are aligned on a single stream (see Fig. 6b and d)^6,26^, (iv) above a second threshold of the Reynolds number, as observed by Zhang et al.^32^, (or also Zhang et al.^5^ in a serpentine channel), the particles start to defocus again due to the strong mixing effect of the secondary flow.

**Figure 7:**
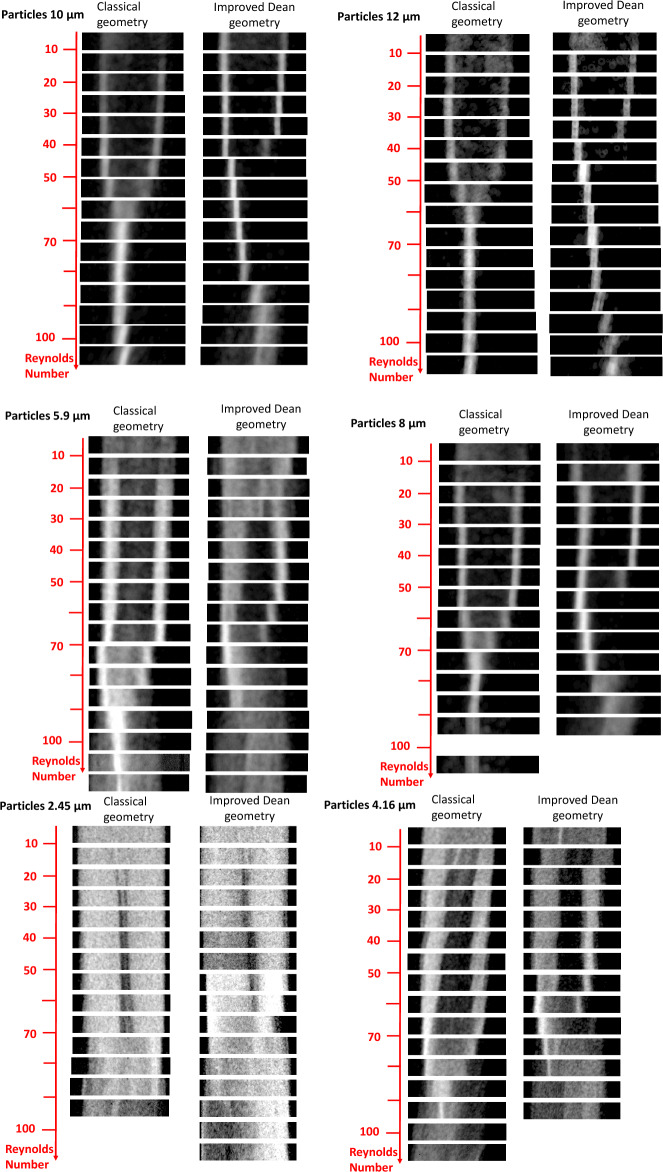
Particle trajectories as a function of the Reynolds number for square wave and modified square wave microchannels and for the different particle sizes. These images were extracted from the mean particle trajectory images (see Fig. 4c) over a section of reduced width.

The observed evolution of the focusing lines with the Reynolds number (see Fig. 6) is very similar to the observations of Di Carlo et al.^1^, Zhang et al.^5^, and Cha et al.^6^, i.e., the two focusing streaks gradually shift toward the channel center. They merge in a single focusing streak in the central region when the Reynolds number exceeds a critical Reynolds number (see Fig. 7). Figure 7 is obtained by always selecting the same section of the mean particle trajectory (i.e., the image inscribed by the rectangular dashed line in Fig. 6c), for different Reynolds numbers and superposing them in a chart on top of each other. These charts are plotted for both geometries and for large, medium, and small particles for comparison. This representation of the mean particle path with increasing Reynolds number allows a good visualization of the different regimes: (i) the lift-dominated regime with two streams of particles, (ii) the Dean drag force-dominated regime with one streams, (iii) the transition between these regimes, and (iv) the absence of focusing at very small Reynolds numbers.

**Figure 8:**
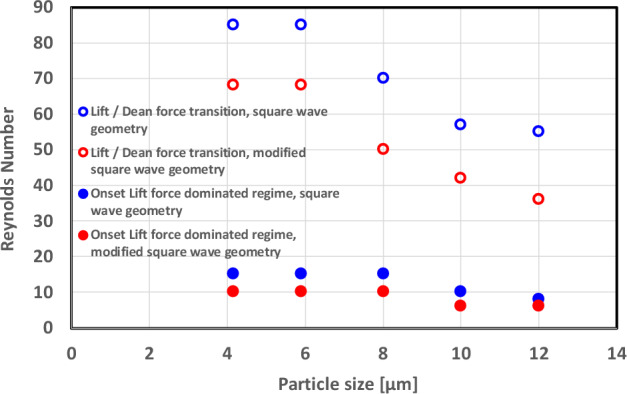
Onset of the lift force-dominated regime (full circle) and critical Reynolds number of the transition between the lift force and Dean vortex-dominated regime (open circle) for the square wave microchannel (blue symbols) and the modified square wave microchannel (red symbols).

The transition from the lift force to the Dean drag force-dominated regime depends on the particle size and geometry (see Figs. 7 and 8). This transition occurs earlier (smaller channel Reynolds numbers) for the modified square wave microchannel than for the square wave microchannel. The location and length of the lift-dominated regime, the Dean-dominated regime, and the transition between these two regimes depend on the particle size, Reynolds number, and microchannel design. They occur earlier for larger particles, which is in agreement with the observations in the literature.

The particle size influences the quality of the focusing. The critical particle blockage ratio used commonly in the literature, $\lambda ={a}_{p}/{D}_{h}>0.07$, is only met for the ${a}_{p}=5.9, 8, 10,$ and 12 µm particles (see Table 1), and indeed, for these particle sizes, the focusing in the lift force-dominated regime and in the Dean drag force-dominated regime is very good. For the 2.45 and 4.16 µm particles, the particle blockage ratio is in the ‘rough’ focusing mode $\sim 0.01 < \lambda <0.07$^62^, and the particles migrate to form a relatively wide particle band (see Fig. 7).

The transition between the lift force-dominated regime and the Dean drag force-dominated regime is qualitatively different for the two geometries. For the square wave microchannel, the transition from two focusing lines to a single focusing line located in the center of the channel occurs gradually by progressively moving the two focusing lines closer to each other with increasing Reynolds numbers until they completely merge. For the modified square wave microchannel, the two focusing lines begins to move closer to each other only when one line gradually disappears in favor of the other. Furthermore, after this sudden merging, the remaining single focusing line continues to move progressively toward the center of the channel.

The Reynolds number of the onset of the lift force-dominated regime as well as the critical Reynolds number where the transition between the lift force and Dean drag force-dominated regime occurs are smaller for the modified square wave microchannel than for the square wave microchannel for all the investigated particle sizes (see Fig. 8). Therefore, it can be concluded that the modified square wave microchannel is more efficient in focusing the particles. In addition, with a large region of Dean drag force dominated regime in the modified square wave microchannel, especially for large particles, the size-based separation mechanism can work effectively; therefore, we can conclude that the modified square wave microchannel is more effective in focusing and separating particles of different sizes.

**Figure 9:**
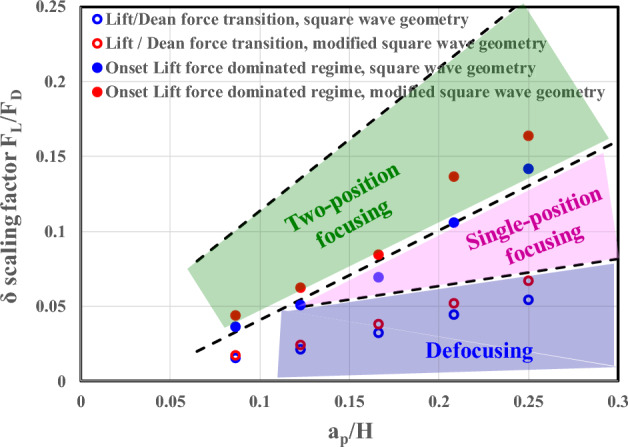
Comparison between the experimental operational map of Zhang et al.^32^ for a symmetric sinusoidal channel and our data for square wave and modified square wave microchannels. Onset of the lift force-dominated regime (full circle), lift force- and Dean vortex-dominated regime (open circle) for the square wave microchannel (blue symbols) and the modified square wave microchannel (red symbols).

An experimental operational map of the inertial focusing pattern was established by Zhang et al.^5^ for a sinusoidal channel. They plotted the scaling factor of the Lift and Dean forces $\frac{{F}_{L}}{{F}_{D}}\sim \frac{{a}_{p}/{D}_{h}}{{De}^{1/2}{\left({D}_{h}/2R\right)}^{3/4}}=\delta$ as a function of (a/H), which is a modified particle blockage ratio $\frac{a}{H}=\left(a/{D}_{h}\right)\times \frac{2}{\left(1+\frac{H}{W}\right)}$. In Fig. 9, their results and our results are plotted on the same graph. Qualitatively, the results are very similar, with similar slopes, but the onsets and transitions between domains are lower in our square wave and modified square wave microchannels than in the sinusoidal channel of Zhang et al.^5^, which may be due to the difference in geometries between their sinusoidal channel and our square wave channels.

### Histograms of particle distribution

To be more quantitative in comparing the efficiency of the two geometries, a histogram of the particle distribution is performed on a cross-section of the channel. These histograms were obtained by individually detecting the particles on images such as the one shown in Fig. 4d. On an area corresponding to a cross-section of the channel times a limited length of the channel (100 µm in our case), each particle present in this area is recognized individually and its center is calculated for the 2000 images of each series. By summing all these data, the histogram and standard deviation of the particles around a mean value can be calculated.

**Figure 10:**
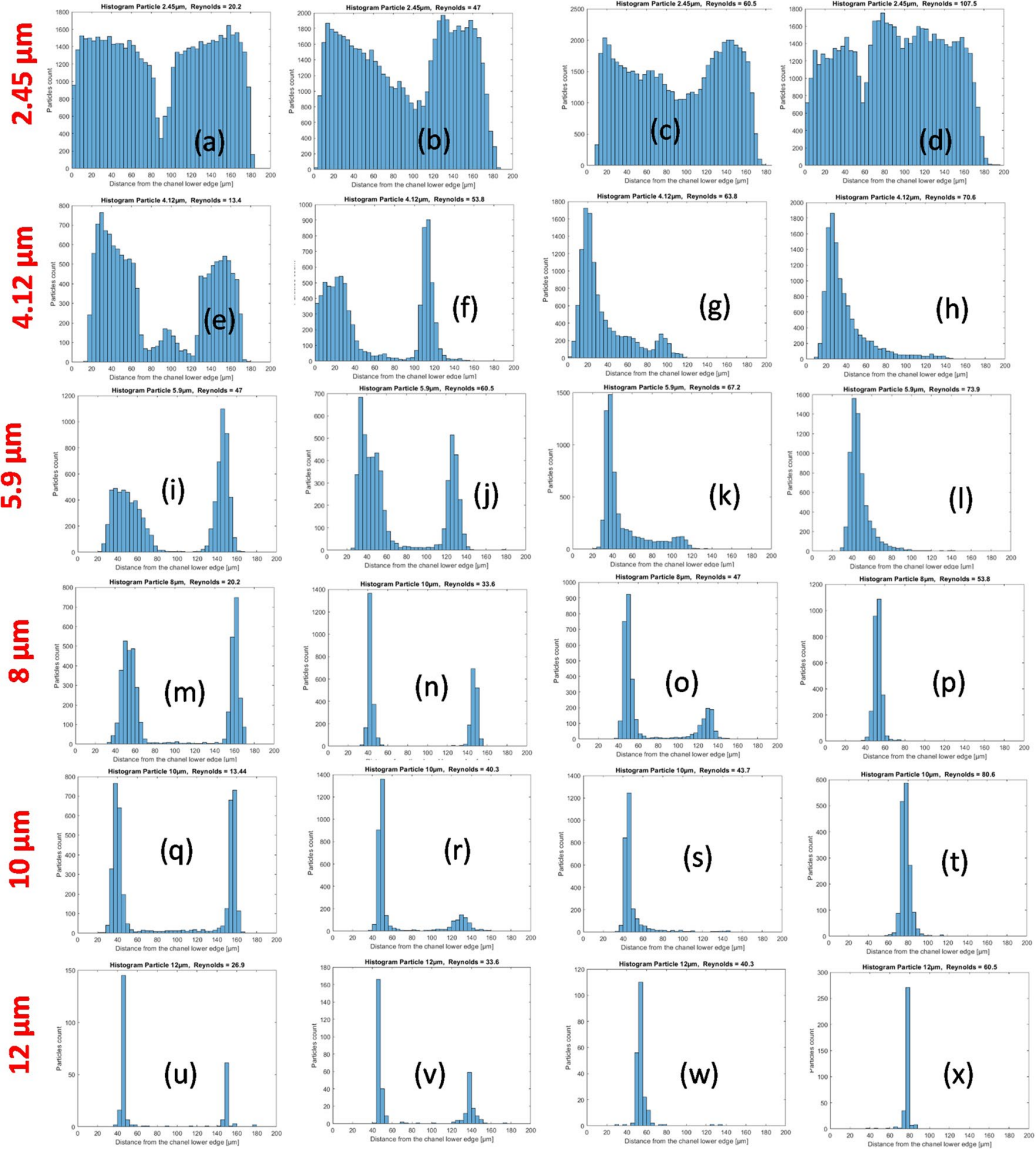
Histogram of the particle distribution on a cross-section of the channel in the case of the modified square wave microchannel. (**a**–**d**) 2.45 µm particles, Reynolds numbers of 20.2, 47.0, 60.5 and, 107.5, respectively; (**e**–**h**) 4.12 µm particles, Reynolds numbers of 13.4, 53.8, 63.8, and 70.6, respectively; (**i**–**l**), 5.9 µm particles, Reynolds numbers of 47, 60.5, 67.2 and, 73.9, respectively; (**m**–**p**) 8 µm particles and Reynolds numbers of 20.2, 33.6, 47 and, 53.8, respectively; (**q**–**t**) 10 µm particles and Reynolds numbers of 13.4, 40.3, 43.7, and 80.6, respectively; (**u**–**x**) 12 µm particles and Reynolds numbers of 26.9, 33.6, 40.3, and 80.5, respectively.

**Figure 11:**
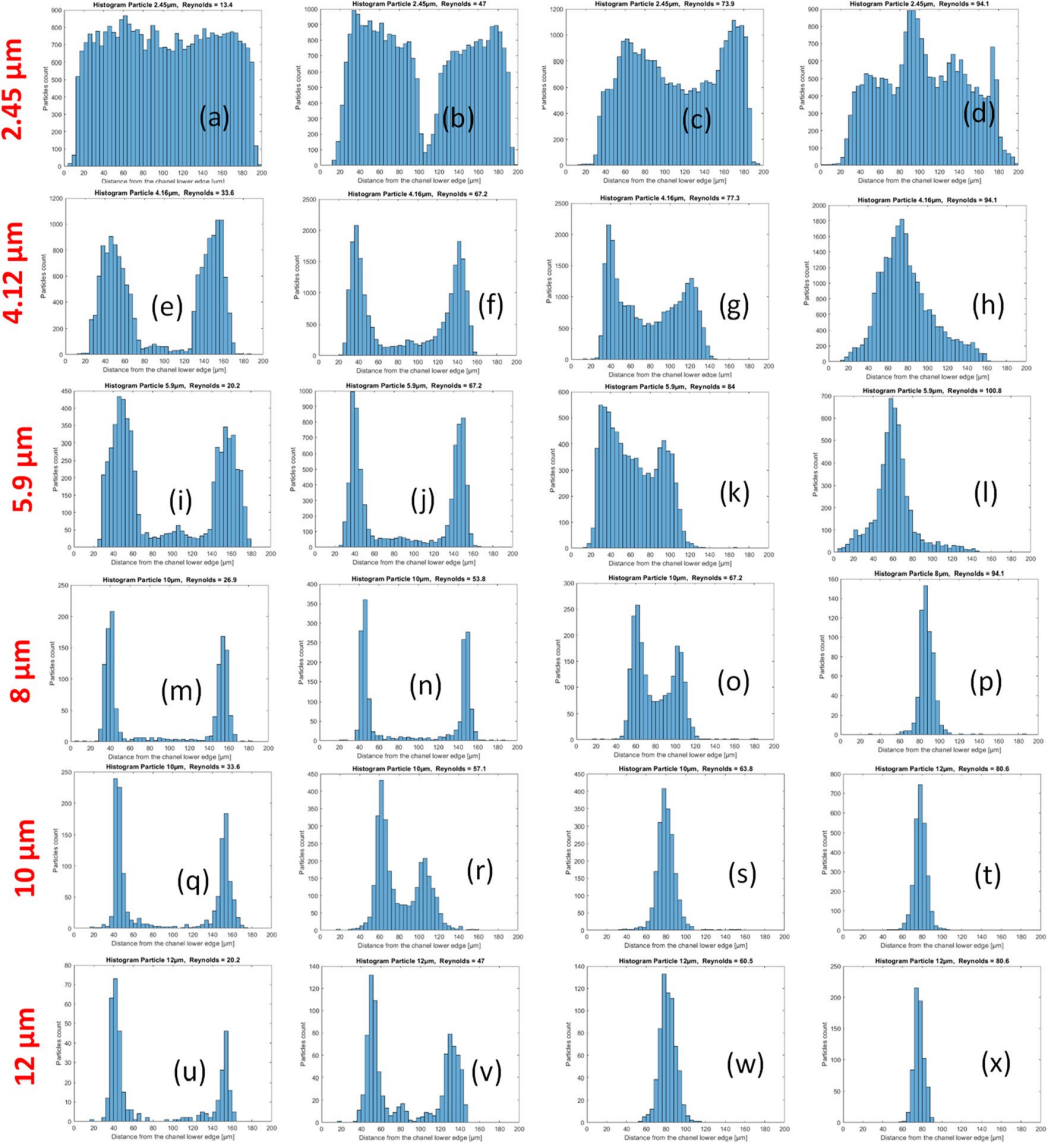
Histogram of the particle distribution on a cross-section of the channel in the case of the square wave microchannel. (**a**–**d**) 2.45 µm particles, Reynolds numbers of 13.4, 47.0, 73.9, and 94.1, respectively; (**e**–**h**) 4.12 µm particles, Reynolds numbers of 33.6, 67.2, 77.3, and 94.1, respectively; (**i**–**l**), 5.9 µm particles, Reynolds numbers of 20.2, 67.2, 94, and 100.8, respectively; (**m**–**p**) 8 µm particles and Reynolds numbers of 26.9, 53.8, 67.2, and 94.1, respectively; (**q**–**t**) 10 µm particles and Reynolds numbers of 33.6, 57.1, 63.8, and 80.6, respectively; (**u**–**x**) 12 µm particles and Reynolds numbers of 20.2, 47, 60.5, and 80.5, respectively.

The particle distribution on a cross-section is represented as histograms in Figs. 10 and 11 for the square and modified square wave microchannels, respectively. For all particle sizes, histograms are in rows and are presented with increasing Reynolds numbers from left to right.

The 2.45 and 4.16 µm particles have a particle blockage ratio of 0.032 and 0.054, respectively, i.e., are in the ‘rough’ focusing’ range, and thus below the generally accepted criterion of 0.07. Therefore, it is not surprising that the particles only partially migrate to form relatively wide particle bands (see Figs. 10 and 11a–h). Particle focusing depends on the particle size, and the optimal focusing is clearly an increasing function of the particle size with excellent focusing for ${a}_{p}=10$ and 12 µm particles.

For each particle size and for both geometries (Figs. 10 and 11), the 2 histograms on the left are in the lift forces-dominated regime. The particles are focused in two streaks, which is typical of the lift force-dominated regime. These two streaks are located about $\frac{1}{4}$ of the channel width from the wall. Most of the histograms in the third column and all of the histograms in the last column (Figs. 10 and 11) are in the Dean drag force-dominated regime, with a characteristic single streak located approximately in the center of the channel.

Below the lift/Dean drag force transition, we observe that the streaks move progressively closer with increasing Reynolds number (and also coming closer while getting closer to the transition), see Fig. 11m–o,q,r, but also Fig. 10q,r,u,v. This seems to be a general feature of the hydrodynamic focusing of particles.

In addition, differences in the lift force/Dean drag force transition also appear between the square wave and modified square wave microchannels. For the square wave microchannel, the two streaks of particles play a symmetrical role in the lift force-dominated regime and are suddenly replaced by a single streak located in the center of the channel after the transition to the Dean drag force-dominated regime. For the modified square wave microchannel, the two streaks do not play the same role. Before the transition, one streak became progressively stronger than the other (see Fig. 10k,o,r,v), and shortly after the transition, the now unique streak (see Fig. 10k,s,w) is located close to the previous location of the stream that was growing stronger before the transition. This unique streak gradually moves from its initial location (about $\frac{1}{4}$ of the channel wall) toward the center of the channel. One streak is absorbed by the other one, until it disappears completely once it enters the Dean drag force-dominated regime.

**Figure 12:**
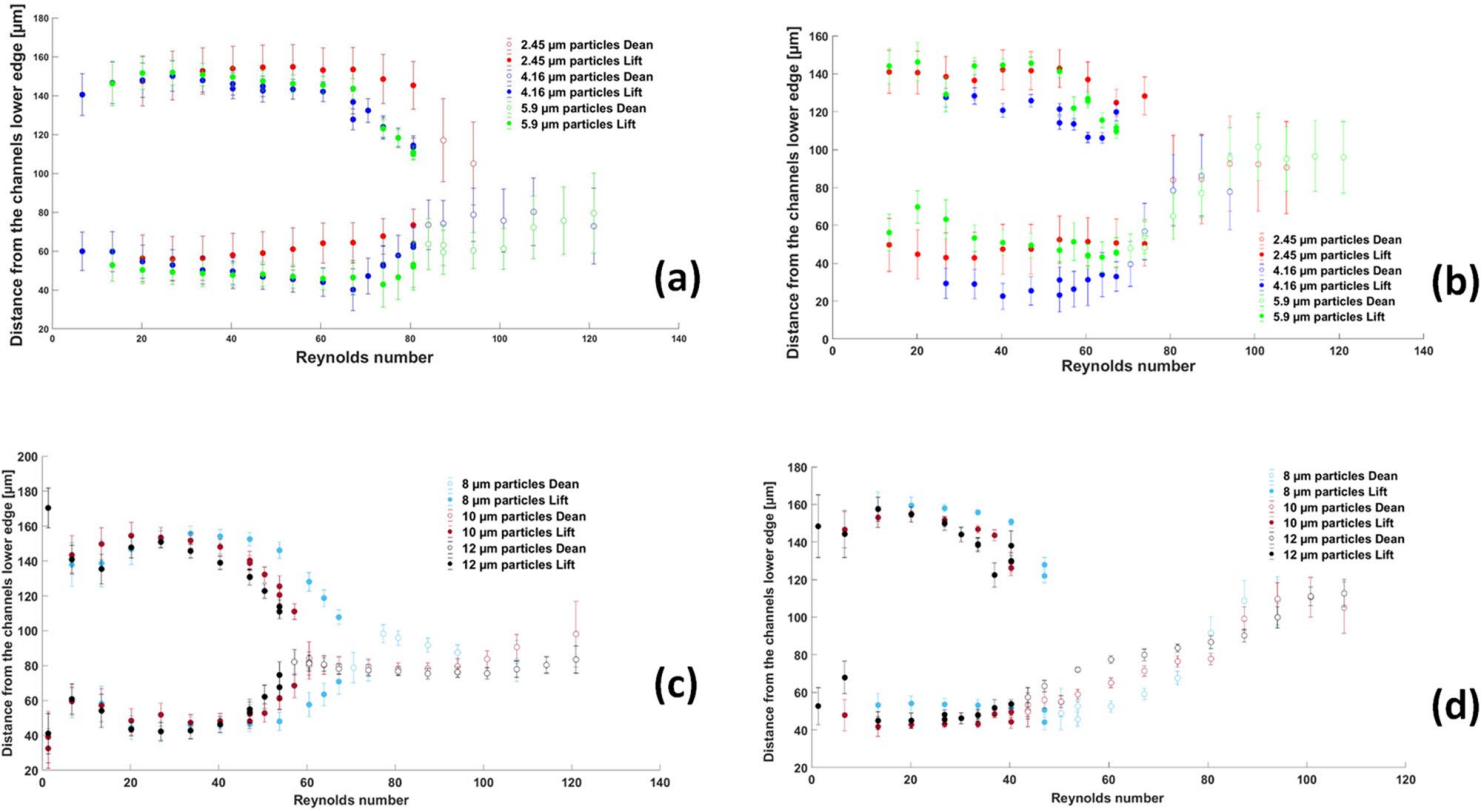
Location of particle streak as a function of Reynolds number. The full symbols (full circles) represent the lift force-dominated regime, and the open symbols (open circles) represent the Dean drag force-dominated regime. (**a**) and (**b**) are the case of small particles (2.45–4.16–5.9 µm) in the square and modified square wave microchannels, respectively, and (**c**) and (**d**) are the case of large particles (8.0–10.0–12.0 µm) in the square wave and modified square wave microchannels, respectively. The standard deviation around the mean value of the particle location is also represented for each point.

This can also be observed in Fig. 12b and d, where the location and standard deviation of the particle streaks are plotted as a function of the Reynolds number for different particle sizes in the modified square wave microchannel. This figure will be further discussed in the next section.

### Streak location dynamics in microchannels

The exact location of the two streaks in the case of the lift force-dominated regime and the single streak in the case of the Dean drag force-dominated regime can be calculated from the particle distribution and is shown in Fig. 12. In the fully developed lift force-dominated regime, the distance between the two particle streaks is about 100 µm, so half of the channel width (see Fig. 12a and c), and therefore, the streak is around 50 µm away from the channel sidewall. In Fig. 12 the transition between the lift force-dominated regime and the Dean drag force-dominated regime shows some interesting features: (i) in the square wave microchannel, the two particle streaks formed in the lift force-dominated regime clearly converge toward each other by reducing the distance between them to half of the distance that these streaks have in the fully developed lift force-dominated regime (i.e. 50 µm compared to 100 µm), (ii) the fluctuations are higher during this transition than in the well-established lift force- or Dean drag force-dominated regime, (iii) in the modified square wave microchannel, the non-axisymmetric nature of the geometry is evident, with the two particle streams having unequal weights (see Figs. 2, 3). In addition, in this modified square wave microchannel, the transition is not symmetric, but looks more like an absorption of the small stream by the stronger one, so that only one particle stream is present in the Dean drag force-dominated regime. This transition is also more abrupt than in the case of the square wave microchannel, with only a limited convergence of the two lift force-induced streams before the transition, (iv) in the square wave microchannel, the particle stream is in the center of the channel immediately after the transition to the Dean drag force-dominated regime, whereas in the modified square wave microchannel, the particle stream converges only slowly toward the center of the channel as the Reynolds number increases. It is noteworthy that for both geometries, the standard deviation around the mean value of the stream position is significantly high for the smallest particle size used in our experiment (2.45 µm). This holds true for both lift force- and Dean drag force-dominated regimes, with a notable reduction in standard deviation when larger particles are employed.

As already observed in Figs. 4 and 5, (i) for both geometries, the transition from the lift force-dominated regime to the Dean drag force-dominated regime depends on the particle size (as already observed by Cha et al.^6^). The Reynolds number at which this transition occurs is a decreasing function of the particle size. (ii) For all particle sizes, the transition occurs earlier in the modified square wave microchannel than in the square wave microchannel.

Confirming the observations by Di Carlo et al.^1^, the equilibrium position in the lift-dominated regime shifts toward the center of the channel with increasing particle size (or particle blockage ratio, $\frac{{a}_{p}}{H}$). The modified square wave microchannel is particularly suitable for particle separation. In the Dean drag force-dominated regime (see Fig. 12b and d), the stream of particles is at a well-defined location for each particle size and is well separated. This location strongly depends on the particle size.

**Figure 13:**
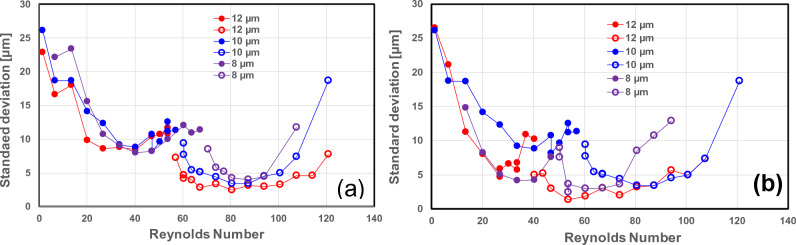
Standard deviation around the mean value of the stream. Solid symbols represent the lift force-dominated regime, open symbols represent the Dean drag force-dominated regime, (**a**) for the square wave microchannel, and (**b**) for the modified square wave microchannel.

The standard deviation around the position of these streaks was also calculated and is represented in Fig. 13 for the different particle sizes and two geometries as a function of the Reynolds number. The fluctuation of the stream depends on the Reynolds number. There is a minimum of fluctuations (minimum standard deviation around the mean value, see Fig. 13) for the lift force- and Dean drag force-dominated regime. At low Reynolds numbers, before the lift force regime is well established, fluctuations in the particle positions around the mean value are high, and these fluctuations are decreasing slowly until they reach a minimum. Similarly, at high Reynolds numbers (Reynolds number above 100), the Dean drag force is unable to concentrate the particles in a narrow stream, and the fluctuations grow with Reynolds number. Cha et al.^6^ made similar observations. As the Reynolds number is further increased, the Dean drag forces become largely dominant over the lift force, and the focusing is no longer good because the Dean drag force becomes too strong and the mixing effects from this secondary flow begin to dominate^6,21,26^. The transition between these two regimes is characterized by an increase in fluctuations before and after the transition. An increase in fluctuations around a transition is a feature often observed in physics and is once again illustrated in the present study (see Fig. 13). Both geometries show very similar behavior with respect to the fluctuation of the stream.

**Figure 14:**
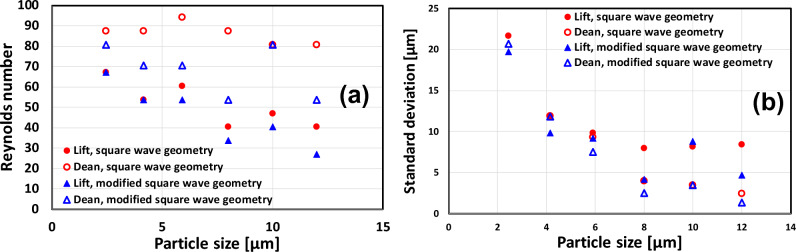
(**a**) Reynolds numbers corresponding to the highest efficiency of lift force and Dean force to focus the particle in one or two lines, respectively, for each particle size and for both geometries, (**b**) corresponding standard deviation.

A last point to be considered is to determine the experimental conditions where the focusing of the particle stream in the Lift force and Dean drag force regimes is the sharpest. For this purpose, the Reynolds numbers and the standard deviation around the mean corresponding to the minimum standard deviation were determined for each particle size, each regime, and both geometries (see Fig. 14a and b, respectively).

Some observations can be made: (i) For large particle sizes (${a}_{p}=8, 10,$ and 12 µm), the sharpest focusing of the particle stream is obtained for a lower Reynolds number than for smaller particle sizes, i.e., for large particles, the focusing in the lift force and the Dean drag force dominated regime are reached for lower flow rates in both geometries. The Reynolds number required for optimal focusing is a decreasing function of the particle size for both flow regimes and both geometries, (ii) the modified square wave microchannel is more efficient at focusing the particles than the square wave microchannel, i.e., good focusing of the particle stream is achieved at lower flow rates This can be observed in the lift force- and Dean drag force-dominated regimes, and (iii) The width of the particle stream is a decreasing function of the particle size (see Fig. 14b) for both geometries and regimes. Focusing is higher in the Dean drag force regime than in the lift force-dominated regime. The focusing of the particle stream is significantly better in the modified square wave microchannel for both regimes, with standard deviations as low as 2 µm for the Dean vortex-dominated regime and 4 µm for the lift force-dominated regime.

## Conclusion

In this study, we conducted a comprehensive investigation of particle focusing in microchannels with square-wave and modified square-wave geometries. The experiments covered a wide range of Reynolds numbers ([6.7 ≤ Re ≤ 107.5]) and particle sizes (2.45 µm to 12 µm), and provided insight into the critical Reynolds numbers for the lift- and Dean force-dominated regimes.

The analysis revealed distinct phases in the evolution of particle focusing with increasing Reynolds number. In the lift-dominated regime, particles focused symmetrically on two streams around the channel center. As the Reynolds number increased, the Dean drag force began to compete with the inertial lift forces, leading to a transition phase. Beyond a critical Reynolds number, the Dean drag force dominated, aligning the particles on a single stream. Further increases in the Reynolds number resulted in defocusing due to strong mixing effects from the secondary flow.

Comparisons between the square-wave and modified square-wave microchannels demonstrated the superior efficiency of the latter in focusing and separating particles of different sizes. The onset of lift dominance and the critical Reynolds number for the transition from lift to Dean drag occurred at lower Reynolds numbers in the modified square wave microchannel for all particle sizes. This efficiency was attributed to a larger region of Dean drag dominance, especially for larger particles, facilitating effective size-based separation. Streak location dynamics analysis revealed the unique characteristics of lift and Dean drag transitions in both geometries. The modified square-wave microchannel exhibited an asymmetric transition, with one particle stream gradually overpowering the other before merging. The equilibrium position in the lift-dominated regime shifted toward the channel center with increasing particle size.

Quantitative evaluation through histograms of particle distribution and standard deviation analysis showed that for both geometries, larger particles exhibited superior focusing in both lift- and Dean drag force-dominated regimes. The modified square-wave microchannel consistently outperformed the square-wave microchannel, achieving sharper particle flow focusing at lower Reynolds numbers. There is an optimal flow regime for the lift force-dominated regime and for the Dean drag force-dominated regime, where the standard deviation around two streams and one stream, respectively, is the smallest, where the separation between streams corresponding to different particle sizes is good. The sweet spot for particle separation is particularly present in the Dean drag force-dominated regime for the modified square wave microchannel, where the location of the streams is highly size dependent and the standard deviation around a mean value is also highly reduced. Fluctuations of the stream(s) are high around the transition from the lift force to the Dean drag force-dominated regimes, which is a common feature in physics.

The results provide valuable insights into microfluidics and particle manipulation techniques in square wave serpentine microchannel, paving the way for improved applications in particle sorting and lab-on-a-chip technologies. Furthermore, as an interesting avenue for future research, the optimization of serpentine microchannels could be explored to improve particle manipulation techniques and provide additional insight into the design parameters that contribute to improved efficiency, footprint, throughput, resolution, and parallelization in microfluidic applications.

## Data Availability

The data supporting the results of this study are available from the corresponding authors upon reasonable request.
